# Predictive values of coronary artery calcium and arterial stiffness for long‐term cardiovascular events in patients with stable coronary artery disease

**DOI:** 10.1002/clc.23955

**Published:** 2022-11-29

**Authors:** Thosaphol Limpijankit, Sutipong Jongjirasiri, Krissada Meemook, Nattawut Unwanatham, Ammarin Thakkinstian, Jiraporn Laothamatas

**Affiliations:** ^1^ Division of Cardiology, Department of Medicine, Faculty of Medicine Ramathibodi Hospital Mahidol University Bangkok Thailand; ^2^ Department of Radiology, Faculty of Medicine, Ramathibodi Hospital Mahidol University Bangkok Thailand; ^3^ Department of Clinical Epidemiology and Biostatistics, Faculty of Medicine Ramathibodi Hospital Mahidol University Bangkok Thailand; ^4^ Faculty of Heath Science Technology Chulabhorn Royal Academy Bangkok Thailand

**Keywords:** arterial stiffness, cardio‐ankle vascular index (CAVI), coronary artery calcium (CAC) score, major adverse cardiovascular events (MACEs)

## Abstract

**Background:**

Subclinical atherosclerosis detected by increased coronary artery calcium (CAC) or arterial stiffness as reflected by cardio‐ankle vascular index (CAVI) has been associated with major adverse cardiovascular events (MACEs). However, comparative data from these two assessments in the same population are still limited.

**Methods:**

From 2005 to 2013, patients with stable coronary artery disease (CAD), both asymptomatic and symptomatic who underwent both coronary computed tomography and CAVI were enrolled and followed for occurrence of MACEs (cardiovascular [CV] death, nonfatal myocardial infarction [MI], and nonfatal stroke) until December 2019. A cause‐specific hazard model was applied to assess the associations of CAC score, and CAVI with long‐term MACEs.

**Results:**

A total of 8687 patients participated. Of them, CAC scores were 0, 1–99, 100–399, and ≥400 in 49.7%, 31.9%, 12.3%, and 6.1%, respectively. Arterial stiffness (CAVI ≥ 9.0) was associated with the magnitude of CAC in 23.8%, 36.3%, 44.5%, and 56.2%, respectively. During an average of 9.9 ± 2.4 years follow‐up, MACEs occurred in 8.0% (95% CI: 7.4%, 8.6%) of subjects. After adjusting for covariables, CAC scores of 100–399 and ≥400, and CAVIs of ≥9.0 were found to independently predict the occurrence of MACEs with the hazard ratios (95% CI) of 1.70 (1.13, 1.98), 1.87 (1.33, 2.63), and 1.27 (1.06, 1.52), respectively. Other risk predictors were hypertension, diabetes mellitus (DM), chronic kidney disease (CKD), aspirin, and statin therapy.

**Conclusions:**

A CAC score ≥100 or a CAVI ≥ 9.0 predicts the long‐term occurrence of MACEs in both asymptomatic and symptomatic patients with stable CAD. These two noninvasive tests can be used as screening tools to guide treatment for the prevention of future CV events.

## 1 INTRODUCTION

Early detection of subclinical atherosclerotic cardiovascular disease (ASCVD) can guide management to prevent myocardial infarction (MI), stroke, and cardiovascular (CV) death.^1^, ^2^ Several ASCVD risk scoring systems (e.g., Framingham and American Heart Association/American College of Cardiology [AHA/ACC] risk scores) have been widely used in clinical practice to predict the 10‐year risk of cardiovascular (CV) events.^3^, ^4^ However, these scores often overestimate the risk of future CV events and do not enable better targeting of individual preventive treatment.^5^

In addition to these risk scores, the accuracy of prediction may be improved by using an arterial stiffness measurement calculated from pulse wave velocity (PWV) or the cardio‐ankle vascular index (CAVI), or noninvasive imaging with coronary computed tomography (CT) scan.^6^, ^7^ CAVI is more widely used in clinical practice than PWV as a surrogate of early atherosclerosis and is independent of blood pressure (BP) changes.^8^ Multiple studies confirm a strong association between CAVI and subclinical coronary artery disease (CAD) detected by coronary CT.^9^, ^10^ Furthermore, CAVI has been shown to enhance traditional risk scores as predictors of CAD and future CV events.^11^

Coronary artery calcium (CAC) measured by coronary CT implies the presence of CAD and is associated with increased risk of CV events.^12^, ^13^, ^14^ In contrast to CT angiography for coronary stenosis, the CAC score provides a reliable, noninvasive index with no use of contrast media while keeping radiation exposure low. Evidence from the multi‐ethnic study of atherosclerosis (MESA) indicates that CAC scoring can significantly improve classification and distinguish patients who are at risk,^15^ and help to guide primary prevention.^16^, ^17^ Thus, CAC scoring provides an adjunctive test, enhancing clinical risk prediction, and more accurately classifying individuals with an intermediate to high ASCVD risk or symptomatic patients with stable CAD who might benefit from primary prevention using aspirin or statins.

An association between arterial stiffness measured by CAVI and CAC scores has been demonstrated in two previous small‐size studies in asymptomatic subjects^9^ and in asymptomatic patients with type 1 diabetes.^18^ They showed that CAVI has a dose‐dependent association with increased CAC score, and can be valuable in identifying individuals at risk for future CV events.^18^ To confirm this relationship in a larger sample size with longer follow‐up, we conducted a study to determine the predictive values of CAC score and arterial stiffness as reflected by CAVI on long‐term cardiovascular (CV) events in both asymptomatic patients with risk factors and symptomatic patients with suspected stable CAD.

## 2 MATERIALS AND METHODS

This was a retrospective cohort study enrolling consecutive patients who underwent coronary CT scan for the assessment of CAD at the Advanced Diagnostic Imaging Center (AIMC), Ramathibodi Hospital, Mahidol University, during November 2005‐November 2013, and were followed up until December 2019. Inclusion criteria were: adults aged >18 years; asymptomatic patients with moderate‐to‐high ASCVD risk,^19^ or patients who had chest symptoms suspicious of CAD, but were clinically stable. Exclusion criteria were severe asthma; high creatinine (>1.5 mg/dl); severe seafood or contrast allergy; history of prior coronary bypass or coronary stenting. The study protocol conformed to the ethical guidelines of the 1975 Declaration of Helsinki and was approved by the Ethics Committee of the Faculty of Medicine, Ramathibodi Hospital, Mahidol University (CAO. MURA2019/758). Written informed consent was obtained from all participants before enrolment in the study.

Subject data recorded included age, sex, risk factors (e.g., smoking, diabetes mellitus [DM], hypertension, and hypercholesterolemia), body mass index (BMI, kg/m^2^), waist circumference (WC), and prior and current medications. Lab data included fasting plasma glucose (FPG), lipid profile, and serum creatinine. DM was defined as overnight FPG ≥ 126 mg/dl or taking antidiabetic medications. Hypertension was defined as systolic BP (SBP) ≥ 140 mmHg and/or diastolic BP (DBP) ≥ 90 mmHg or taking anti‐hypertensive medications. Smoking was classified as current smoking, ex‐smoking more than 1 month, or never smoked. Chronic kidney disease (CKD) was defined as an estimated glomerular filtration rate (eGFR) <60 ml/min/1.73 m^2^. The eGFR was calculated using the CKD Epidemiology Collaboration (CKD‐EPI) equations.^20^

### 2.1 Determination of cardio‐ankle vascular index

**Figure 1:**
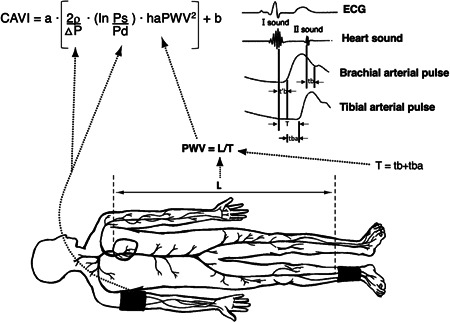
Schematic illustration of cardio‐ankle vascular index (CAVI) measurement. DP, Ps‐Pd; ECG, electrocardiogram; haPWV, heart ankle pulse wave velocity; Ps, systolic blood pressure; Pd, diastolic blood pressure; ρ, blood density; tba, time between rise in brachial pulse wave and rise in ankle pulse wave; tb, time between closing sound of aortic valve and notch in brachial pulse wave; t'b, time between opening sound of aortic valve and rise in brachial pulse wave; a and b, scale conversion constants. (Modified from Shirai K, Utino J, Otsuka K et al.^21^)

To measure arterial stiffness, our study team planned and designed a protocol to perform CAVI on the same day of the coronary CT study. A participant's CAVI was determined using a Vasera VS‐1000 vascular screening system (Fukuda Denshi),^21^ that was used to measure ankle‐brachial index (ABI) for diagnosis of peripheral arterial disease. In brief, patients in the supine position had pressure cuffs applied bilaterally to upper arms and ankles. After resting for 10 min, the machine monitored the electrocardiogram and phonocardiogram to measure PWV from heart to ankle. This was obtained by measuring the distance between the aortic valve to the ankle (L) and dividing by the time for the pulse wave to propagate from the aortic valve to the ankle (T). CAVI is calculated from this PWV and BP using the following equation: CAVI = *a*{(2*ρ*/Δ*P*) x ln(Ps/Pd) x haPWV^2^}+*b*, where Ps and Pd are the SBP and DBP, respectively; PWV is the PWV between the heart and ankle; *ρ* is blood density; Δ*P* is Ps‐Pd; *a* and *b* are scale conversion constants (Figure 1).^21^ The Index was calculated from the heart‐ankle pulse wave velocity (haPWV) adjusted for BP and based on stiffness parameters. In this study, the mean value of right and left CAVIs was used for analyses. According to the manufacturer, values <8.0 are considered as normal, 8.0 to <9.0 as borderline, and ≥9.0 as high, suggestive of the presence of arteriosclerosis and predictive of CV risk.^22^ We used the CAVI as a categorical rather than a continuous variable. A cut‐off of CAVI ≥9.0 is generally considered as significant arterial stiffness and used to predict CV risk.^23^

### 2.2 Coronary CT

The multidetector CT (MDCT) scans done during the study used either a 64‐slice CT scanner (Somatom Sensation 64 eco, Siemens) or a 320‐slice CT scanner (Aquilion ONE, Toshiba) before and after 2008, respectively. Two coronary CT scan findings used in this analysis were (i) CAC score and (ii) degree and extent of stenosis by CCTA. The CAC score was calculated according to the Agatston method using a commercially available external workstation (Vitrea fx 3.0.1, Vital Images). Total CAC score was calculated as the sum of the individual lesion scores in all coronary arteries and then stratified into four groups: 0, no identifiable plaque; 1–99, mild atherosclerotic plaque; 100–399; moderate atherosclerotic plaque; and ≥400, extensive atherosclerotic plaque.

Degree of coronary stenosis was evaluated after injecting 70–90 ml of radiocontrast (Ultravist 370 mgI/ml, Bayer Healthcare Pharmaceuticals) via the right basilic vein through an 18‐gauge intravenous catheter followed by a 20 ml saline flush at a flow rate of 5 ml/sec. Automated bolus tracking was used to synchronize the arrival of the contrast media and the scan. After a 4‐s delay, images were obtained during an inspiratory breath hold of 5–10 s. Three‐dimensional reconstruction and cross‐sectional imaging measurements were performed; and stenoses were classified as normal (0%), mild (1–<25%), mild‐moderate (25–<50%), moderately severe (50–<75%), or severe (≥75%). In this study, we reclassified into three groups: no CAD (0% stenosis), nonobstructive CAD (1%–49% stenosis), and obstructive CAD ( ≥ 50% stenosis).

### 2.3 Data collection

From each subject, the following data were recorded: age, sex, risk factors (e.g., smoking, DM, hypertension, and hypercholesterolemia), BMI, WC, and prior and current medications during the follow‐up period including aspirin and statin. Also recorded were blood tests (e.g., FPG, lipid profiles, serum creatinine, and eGFR), ABI, CAVI, and full report of coronary CT scan. The data were computerized by two independent healthcare personnel (i.e., the trained catheterization laboratory nurses or AIMC staff). Data were double‐checked for inconsistency and readjusted for accuracy. Finally, electronic databases were exported into Excel spreadsheets for statistical analysis.

### 2.4 Treatment after coronary CTA

Patients were scheduled to see a cardiologist or internist with the official report of CCTA (i.e., degree of CACS, and severity and location of coronary stenoses), CAVI and baseline data. After completion of this evaluation, patients were managed based upon physician's discretion including aspirin, statin, and risk factor modification. Some patients might have additional tests such as a cardiac stress test, and/or an invasive coronary angiography with possible revascularization (either percutaneous coronary intervention [PCI] or coronary artery bypass surgery [CABG]).

### 2.5 Long‐term clinical follow‐up

Clinical outcomes were retrieved after performing the coronary CT scan until December 2019 by accessing data from three sources: the electronic medical records, Information Technology (IT) division of Ramathibodi Hospital; 43‐file data from the strategy and planning division, Office of the Permanent Secretary Ministry of Public Health; the Information and Communication Technology (ICT) of Ministry of Public Health and Center Office for Healthcare Information.

The clinical outcomes of interest were major adverse cardiovascular events (MACEs), including CV death, nonfatal MI, or nonfatal stroke. If a subject had more than one of these CV events, only the first event was counted. CV deaths were classified as either a CAD death or a stroke death, confirmed by death certificates from the National Statistics Office, Ministry of Interior. CAD was defined as the presence of at least one of the following: angina, acute coronary syndrome, acute MI, a significant (>70% diameter stenosis) lesion on coronary angiography, revascularization (PCI or CABG), or documented myocardial ischemia during exercise testing. MI was defined as an increase in cardiac troponin (cTn) plus one of the following: (1) evidence of prolonged ischemia as demonstrated by chest pain longer than 20 min; (2) ischemic ST‐segment changes or new pathological Q waves; (3) angiographic evidence of coronary occlusion or no‐reflow/slow flow; (4) imaging evidence of a new loss of viable myocardium or new regional wall motion abnormality. Stroke was defined as a history of ischemic or hemorrhagic stroke, or a transient ischemic attack (TIA), documented by CT or MRI.

### 2.6 Statistical analysis

Baseline characteristics were summarized as mean ± standard deviation (SD) and percentage for continuous and categorical variables, respectively. These were then compared among CAC score groups using one‐way analysis of variance, quartile regression, or *χ* ^2^ test as appropriate. The incidence of MACE was estimated along with its range estimation (i.e., 95% confidence interval [CI]). To evaluate the relationships of CAC score and CAVI to MACEs, a competing risk with subdistribution hazard model was applied to estimate a cumulative incidence function (CIF) considering other causes of death as competing risk events. A multivariate cause‐specific Cox hazard (CSH) regression was applied to assess if CAC score, coronary stenosis, CAVI, and/or other significant risk factors were associated with a MACE (and not another cause of death as a competing risk) with the following steps: Frist, a univariate analysis was applied by fitting each of the risk factors in the CSH model. Second, risk factors whose *p* value was less than .10 from the univariate analysis were simultaneously included in the multivariate CSH model. A backward elimination was applied by eliminating each of the risk factors from the model. A likelihood ratio test was used to keep only significant (*p* < .05) risk factors, as well as CAC score and CAVI in the final model. Hazard ratio (HR) along with its 95% CI for each risk factor was then estimated and reported.

All analyses were performed using STATA 17.0. (Stata Statistical Software: Release 17; StataCorp). *p* values of less than .05 were considered as statistically significant.

## 3 RESULTS

A total of 8687 patients participated in the study; their mean age at enrolment was 59.0 ± 8.4 years, 63.3% were females, and the mean BMI was 24.9 ± 3.6 kg/m^2^. Atherosclerotic risk factors included hypercholesterolemia in 52.5%, hypertension in 42.3%, current or ex‐smoking in 13.8%, DM in 13.7%, and CKD in 7.2%. The mean CAVI was 8.9 ± 2.2, with CAVI ≥ 9 in 36.9%. Baseline blood test results are shown in Supporting Information: Table 1. Most patients in our cohort had either normal coronary arteries (40.2%) or nonobstructive CAD (39.3%), while 20.6% had obstructive CAD (≥50% stenosis in any coronary artery). Overall, 13.7% of patients had one‐vessel disease, 4.8% had two‐vessel disease, 2.8% had three‐vessel disease, and 1.1% had left main disease. The distribution of CAC scores was 0 in 49.6%, 1–99 in 31.9%, 100–399 in 12.4%, and ≥400 in 6.1%.

Most patients were initially managed with medications (*n* = 5855 (67.4%)]. Only a minority of them were investigated further with cardiac stress test (*n* = 35 (0.4%)] and/or underwent coronary angiography (*n* = 226 [2.6%]), and revascularization (*n* = 139 [1.6%]). Most patients had never received prior medications, but they were prescribed after CCTA or occurrence of later MACEs, which resulted in percentages of statin and aspirin therapy increased significantly from 9.4% to 67.4% and 0.5% to 31.5%, respectively. During the follow‐up period, almost one‐third and two‐thirds of subjects were being treated with an antiplatelet agent and statin, respectively (Supporting Information: Table 1).

### 3.1 Association between CAVI and CAC score

The relationship of baseline clinical characteristics, CAVI, and coronary CTA findings to CAC scores are summarized in Table 1. CAVI ≥ 9.0 was associated, in a dose‐dependent manner, with CAC scores, degree and number of coronary stenoses, and LM involvement. Patients with higher CAC scores were more likely to be older, male, and current/ex‐smokers, with greater BMIs and more frequent CV risk factors (i.e., hypertension, DM, CKD). Low HDL‐C level (<40 mg/dl) was more frequent in those with higher CAC scores, and high LDL levels (≥100 mg/dl) were less frequent in those with higher CAC scores. Hyperuricemia was found more frequently in subjects with higher CAC scores. Triglyceride and abnormal WC were not consistently associated with the magnitude of CAC score.

**Table 1:** Baseline clinical, CAVI, and coronary CT findings characteristics, grouped by CAC score

Characteristic	CAC score				*p* value
	0	1‐99	100‐399	≥400	
	(*n* = 4316)	(*n* = 2772)	(*n* = 1072)	(*n* = 527)	
CAVI, %					
≥9	27.4	41.4	50.7	62.2	<.001
<9	72.3	58.6	49.3	37.8	
Degree of stenosis, %					
≥50%	4.3	19.4	57.5	83.5	<.001
1− <50%	29.3	59.6	39.2	15.7	
0%	66.4	21.0	3.3	0.8	
Number of stenotic vessels, %					
3‐vessels	0.05	0.8	5.9	29.6	<.001
2‐vessels	0.3	3.0	15.4	28.5	
1‐vessel	5.0	16.2	36.0	25.2	
None	94.6	80.0	42.7	16.7	
LM > 50%, %	0.1	0.5	2.5	8.7	<.001
Age (years), %					
≥60	32.9	55.0	69.8	81.6	<.001
≥45–<60	60.5	42.8	30.0	18.2	
<45	6.6	2.2	0.2	0.2	
Sex, %					
Male	26.1	42.3	51.8	63.9	<.001
BMI ≥ 23, %	64.9	73.0	71.7	75.0	<.001
Abnormal waist size, %	49.0	51.2	49.4	52.0	.227
Current/Ex‐smoker, %	9.9	15.4	19.6	25.8	<.001
HT, %	51.2	70.2	82.8	86.7	<.001
DM, %	19.6	27.7	35.5	42.6	<.001
CKD (eGFR < 60), %	4.2	7.7	12.9	17.8	<.001
LDL ≥ 100 mg/dL, %	83.6	79.5	76.3	66.7	<.001
HDL‐C < 40 mg/dL, %	12.9	17.3	18.9	21.8	<.001
Triglyceride ≥ 150 mg/dL, %	23.7	28.7	29.3	26.4	<.001
Uric acid ≥ 7 mg/dL, %	10.3	13.5	20.4	20.4	<.001
Concurrent treatment, %					
Aspirin	17.0	32.5	46.8	53.1	<.001
Statin	60.0	71.7	79.4	79.9	<.001

Abbreviations: BMI, body mass index (kg/m^2^); CAC, coronary artery calcium; CAVI, cardio‐ankle vascular index; CKD, chronic kidney disease (eGFR, estimated glomerular filtration rate (ml/min/1.73 m^2^); CT, computed tomography; DM, diabetes mellitus; HT, hypertension; HDL‐C, high‐density lipoprotein‐cholesterol; LDL‐C, low‐density lipoprotein‐cholesterol; LM, left main.

### 3.2 Clinical predictors of long‐term MACEs

Patients were followed for a mean of 9.9 ± 2.4 years. A MACEs occurred in 692 of 8687 subjects, with an incidence (95% CI) of 8.0% (7.4%, 8.6%). These events comprised of CV death in 0.7%, nonfatal MI in 2.3%, and nonfatal stroke in 6.0%. The magnitude of CAC score was associated in a dose‐dependent manner with the frequency of MACEs (Supporting Information: Table 2); CAVI ≥ 9 was also directly related to the frequency of MACEs (Supporting Information: Table 3).

**Figure 2:**
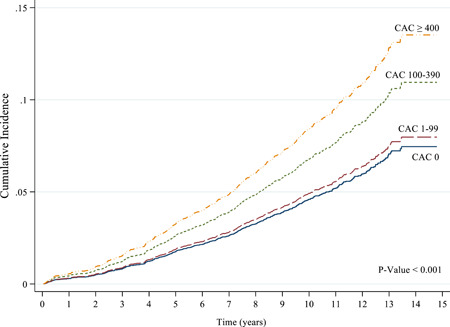
Kaplan–Meier for major adverse cardiovascular events (MACEs) by degrees of coronary artery calcium (CAC) scores

**Figure 3:**
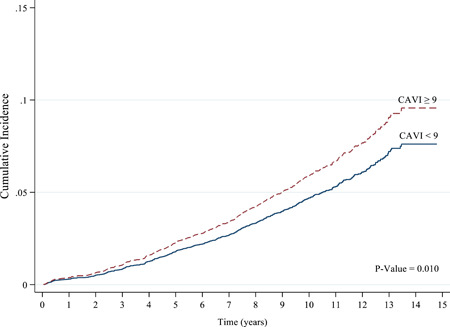
Kaplan–Meier for major adverse cardiovascular events (MACEs) by cardio‐ankle vascular index (CAVI) cut‐off of 9.0

CIF curves were constructed using CAC score and CAVI grouping. These suggested that the probability of MACEs occurrence was significantly higher in the patients with CAC scores of 100–399 and ≥400 (*p* < .001; Figure 2), and with CAVI ≥ 9.0 (*p* = .010; Figure 3). Using these two predictors, differences between groups were visible as early as 2 years postenrolment and persisted through the follow‐up period.

Univariate analysis showed that CAC score, degree of stenosis and number of stenotic vessels, left main disease, and CAVI ≥ 9 were each significantly associated with the occurrence of MACEs (Table 2). In addition, 10 other clinical risk factors were also significantly associated with MACEs (age ≥60, male, BMI ≥ 23, current/ex‐smoker, hypertension, DM, CKD, hyperuricemia [≥7.0], and concurrent aspirin and statin therapy). Higher HDL (≥40) and higher LDL (≥100) levels were found to have preventive effect on MACEs. These counter‐intuitive findings of LDL‐C are due to the inclusion of subjects taking statins in the analysis.

**Table 2:** Subject characteristics, grouped by MACEs and other causes of death: A univariate cause‐specific hazard model

Characteristic	MACEs				Other causes of death			
	Incidence/1000/year	HR	95%CI	*p* value	Incidence/1000/year	HR	95%CI	*p* value
CAC score								
≥400	22.44	4.62	(3.65, 5.84)	<.001	4.94	2.30	(1.48, 3.56)	<.001
100–399	14.61	2.99	(2.42, 3.69)	<.001	6.73	3.14	(2.30, 4.28)	<.001
1‐99	8.06	1.63	(1.35, 1.96)	<.001	3.26	1.49	(1.12, 1.98)	.007
0	5.00	1			2.22	1		
CAVI								
≥9	12.39	2.02	(1.74, 2.36)	<.001	4.58	1.70	(1.34, 2.16)	<.001
<9	5.95	1			2.59	1		
Degree of stenosis								
≥50%	14.03	2.71	(2.23, 3.29)	<.001	4.72	2.01	(1.48, 2.73)	<.001
1–<50%	8.38	1.57	(1.31, 1.88)	<.001	3.54	1.45	(1.11, 1.90)	.007
0%	5.24	1			2.36	1		
Number of stenotic vessels								
3‐vessels	23.77	3.60	(2.68, 4.85)	<.001	4.41	1.54	(0.82, 2.90)	.182
2‐vessels	16.63	2.45	(1.88, 3.20)	<.001	6.93	2.36	(1.58, 3.52)	<.001
1‐vessel	11.39	1.67	(1.37, 2.03)	<.001	3.94	1.33	(0.96, 1.84)	.087
None	6.93	1			3.01	1		
LM disease (>50%)								
Yes	26.82	3.47	(2.22, 5.41)	<.001	9.46	3.02	(1.49, 6.09)	.002
No	8.06	1			3.26	1		
Age (years)								
≥60	12.63	3.87	(2.28, 6.59)	<.001	4.98	3.63	(1.61, 8.17)	.002
≥45–<60	4.68	1.40	(0.81, 2.40)	.225	1.95	1.38	(0.60, 3.14)	.449
<45	3.46	1			1.47	1		
Sex								
Male	10.32	1.47	(1.27, 1.70)	<.001	4.53	1.72	(1.38, 2.15)	<.001
Female	6.96	1			2.59	1		
BMI								
≥23	8.64	1.20	(1.02, 1.41)	.028	3.30	0.98	(0.77, 1.24)	.857
<23	7.22	1			3.37	1		
Waist size								
Abnormal	8.59	1.13	(0.98, 1.30)	.098	3.47	1.14	(0.91, 1.43)	.251
Normal	7.84	1			3.17	1		
Smoking status								
Current/Ex‐smoker	10.98	1.40	(1.16, 1.68)	<.001	5.39	1.79	(1.37, 2.33)	<.001
Never smoked	7.77	1			2.95	1		
HT								
Yes	11.36	3.80	(3.09, 4.68)	<.001	3.81	1.53	(1.20, 1.97)	.001
No	3.00	1			2.47	1		
DM								
Yes	14.25	2.32	(2.01, 2.68)	<.001	4.57	1.58	(1.25, 1.99)	<.001
No	6.18	1			2.88	1		
CKD								
eGFR < 60	20.68	2.88	(2.38, 3.49)	<.001	9.60	3.46	(2.62, 4.56)	<.001
eGFR ≥ 60	7.31	1			2.83	1		
LDL‐C								
≥100	7.52	0.66	(0.56, 0.79)	<.001	3.03	0.64	(0.49, 0.83)	.001
<100	11.24	1			4.69	1		
HDL‐C								
≥40	7.77	0.77	(0.64, 0.92)	.005	2.97	0.60	(0.46, 0.78)	<.001
<40	10.46	1			5.19	1		
Triglyceride								
≥150	9.19	1.17	(0.99, 1.38)	.066	3.11	0.90	(0.69, 1.18)	.441
<150	7.89	1			3.46	1		
Uric acid								
≥7	12.81	1.69	(1.40, 2.04)	<.001	4.95	1.58	(1.18, 2.12)	.002
<7	7.60	1			3.14	1		
Concurrent treatment, %								
Aspirin								
Yes	20.01	5.16	(4.44, 6.00)	<.001	2.06	0.54	(0.40, 0.72)	<.001
No	3.96	1			3.81	1		
Statin								
Yes	11.03	4.09	(3.26, 5.11)	<.001	2.23	0.41	(0.33, 0.51)	<.001
No	2.76	1			5.53	1		

Abbreviations: BMI, body mass index; CAC, coronary artery calcium; CAVI, cardio‐ankle vascular index; CKD, chronic kidney disease (eGFR, estimated glomerular filtration rate (ml/min/1.73 m^2^); CT, computed tomography; DM, diabetes mellitus; HT, hypertension; HDL‐C, high‐density lipoprotein‐cholesterol; LDL‐C, low‐density lipoprotein‐cholesterol; LM, left main; MACEs, major adverse cardiovascular events (cardiovascular death, nonfatal MI, or nonfatal stroke).

A multivariate CSH model revealed that seven clinical risk factors were significantly associated with MACE, see Table 3. Adding each of the CAC scores and CAVI in this CSH model could significantly improve in the prediction of MACE occurrence (LR tests, *p* < .001 for both). This could be interpreted that after adjusting for these covariables using a multivariate CSH regression model, patients with CAC scores of 100–399 and ≥400 were found to be 1.70 (1.13, 1.98) and 1.87 (1.33, 2.63) times more likely, respectively, to develop MACEs than patients with a CAC score of zero (Table 3). Patients with CAVI ≥ 9.0 were 1.27 (1.06, 1.52) times more likely, respectively to develop MACEs than those with lower CAVIs. Importantly, the risk predictor of CAVI ≥ 9.0 was more pronounced in the subgroup analysis of patients with CAC score <100. In these patients with noncalcified plaques or CAC scores <100, CAVI ≥ 9.0 were 1.36 (1.09, 1.69) times more likely to develop MACEs than those with lower CAVIs.

**Table 3:** Predictive value of variables for occurrence of MACEs and other causes of death: A multivariate cause‐specific hazard model

Variable	MACEs		Other causes of death	
	HR (95%CI)	*p* value	HR (95%CI)	*p* value
CACs				
≥400	1.87 (1.33, 2.63)	<.001	1.89 (1.03, 3.47)	.039
100–399	1.70 (1.13, 1.98)	.005	2.98 (1.88, 4.73)	<.001
1–99	1.14 (0.84, 1.36)	.570	1.48 (1,04, 2.11)	.028
0	1		1	
CAVI				
≥9	1.27 (1.06, 1.52)	.010	1.35 (1.02, 1.79)	.035
<9	1		1	
Degree of stenosis				
≥50%	0.86 (0.65, 1.14)	.350	1.18 (0.75, 1.85)	.484
1–<50%	0.89 (0.70, 1.13)	.300	1.02 (0.72, 1.44)	.923
0%	1		1	
HDL‐C				
≥40	0.94 (0.77, 1.15)	.552	0.72 (0.54, 0.98)	.038
<40	1		1	
Age (years)				
≥60	1.69 (0.85, 3.39)	.137		
≥45–<60	1.08 (0.54, 2.17)	.819		
<45	1			
CKD				
eGFR < 60	1.36 (1.05, 1.75)	.019	2.74 (1.88, 4.00)	<.001
eGFR ≥ 60	1		1	
HT				
Yes	1.63 (1.25, 2.13)	<.001		
No	1			
DM				
Yes	1.27 (1.07, 1.52)	.008		
No	1			
Smoking status				
Current/Ex‐smoker			1.60 (1.16, 2.19)	.004
Never smoked			1	
Concurrent treatment, %				
Aspirin				
Yes	3.10 (2.50, 3.84)	.019	0.51 (0.35, 0.75)	.001
No	1		1	
Statin				
Yes	1.73 (1.30, 2.32)	<.001	0.43 (0.33, 0.58)	<.001
No	1		1	

Abbreviations: BMI, body mass index; CAC, coronary artery calcium; CAVI, cardio‐ankle vascular index; CKD, chronic kidney disease (eGFR, estimated glomerular filtration rate (ml/min/1.73 m2); CT, computed tomography; DM, diabetes mellitus; HT, hypertension; HDL‐C, high‐density lipoprotein‐cholesterol; LDL‐C, low‐density lipoprotein‐cholesterol; LM, left main; MACEs, major adverse cardiovascular events (cardiovascular death, nonfatal MI, or nonfatal stroke).

Interestingly, obstructive (degree of stenosis ≥50%) CAD was not found to be a significant predictor of MACEs. Other significant risk factors included age ≥60, hypertension, DM, and CKD, and concurrent aspirin and statin therapy. CAC scores of 100–399 and CAVIs ≥9.0 were also significantly associated with other causes of death along with other factors such as CKD, and current or ex‐smoking.

## 4 DISCUSSION

This cohort was one of the largest yet studied with arterial calcification and arterial stiffness assessed by CAC score and CAVI, respectively, as risk predictors of subsequent MACEs in patients with suspected stable CAD. Our findings suggested that patients with CAC scores of 100–399 and ≥400 were at about 1.70‐ and 1.9‐folds higher risk of developing MACEs than patients with a CAC score of zero. Stenosis ≥50% in coronary arteries was found to not be a significant predictor. Patients with CAVIs ≥9.0 were at 1.27‐folds higher risk of developing MACEs than patients with CAVIs <9.

We included both asymptomatic patients with moderate to high ASCVD risk and symptomatic stable CAD patients with the following reasons: First, to reflect the real practice situation for serving the need of these patients who want to know whether they have subclinical CAD. Second, to help physicians to reclassify patients' risks and third, to expand the spectrum of disease severity. The uniqueness of our study protocol was to demonstrate the comparative data between CAC and CAVI whether they improve risk prediction of long‐term MACEs in stable CAD patients with all stages of atherosclerosis, from no plaque with endothelial dysfunction to fibrofatty (or noncalcified) plaques to calcified plaques. CAC score is well established as a predictor of future MACEs and widely used in risk stratification for primary prevention and treatment.^17^, ^24^, ^25^, ^26^ However, individuals who have a CAC score of zero or <100 may have a silent noncalcified plaque which could develop and rupture later, resulting in symptomatic CAD with MI, stroke, or sudden death.^27^ In our study, the percentage of patients who had CAC scores of 0 or <100 was 49.7% and 31.9%, respectively, forming most of our cohort. In these non‐ or minimally calcified plaque patients, CAVI, as an early physiological surrogate marker of atherosclerosis,^28^ may provide a beneficial role in filling this gap and allow prediction of CV events.

After considering traditional risk factors such as old age, DM, hypertension, CKD, and concurrent aspirin and statin therapy, CAVIs and CAC scores were shown to be independently predictive of long‐term MACEs. We also found that there was a dose‐dependent association between these two measurements, although the existence of calcification was more predictive of MACEs than was arterial stiffness. Both CAC scores ≥100 and CAVIs ≥9.0 were independent risk predictors of long‐term MACEs in asymptomatic patients with risk factors or symptomatic patients with suspected stable CAD.

In this study, we used the CAVI as a categorical rather than a continuous variable. From the previous literature, there may be some controversy as to the optimal cut‐off value for discriminating CV risk.^7^, ^29^ However, a CAVI ≥ 9 is generally considered high and to represent the presence of atherosclerosis and to predict CV risk.^7^, ^18^, ^30^ As expected, CAVIs ≥ 9 was more frequent in our patients who developed MACEs than were CAVI < 9. After adjusting for other confounding factors, CAVIs ≥9 remained as one of the independent risk predictors of CV events, although it was not as strong as the CAC score. Our study adds further information about the benefit of CAVI in subgroup patients with CAC score <100. In these patients with noncalcified plaque or CAC score <100, CAVI ≥ 9 can improve risk prediction of future MACEs. Previous studies have shown an association of both arterial stiffness and coronary CT scan findings (both CAC score and degree of stenosis) with CAD.^9^, ^11^, ^31^, ^32^ These assessments can predict long‐term CV events better than ASCVD risk scores and help to guide primary prevention. However, CAVI has been studied and used mostly in Asian countries (especially Japan) along with a few European countries.^33^, ^34^ Importantly, CAVI is nonspecific and can be increased in older ages, males, uncontrolled hypertension, etcetera.^35^, ^36^ Therefore, it should be used to guide preventive treatment in subjects who have at least an intermediate probability of CAD or in suspected CAD patients with multiple risk factors. Currently, CAVI is not widely used in Western countries and has not been recommended in any guidelines. However, this noninvasive simple test might be useful as an adjunctive tool to guide treatment and prevention after more evidence accumulates.^8^

Contrary to CAVI, the CAC score is more widely used and studies support its use as a screening test to guide treatment in both symptomatic and asymptomatic patients.^37^, ^38^, ^39^ As for a process of model selection, our findings support the hypothesis that adding high CAC score (≥100) into the model which contained only clinical risk factors could increase the magnitude of MACE risk beyond that obtained from clinical risk factors alone. Similar to the MESA findings, participants with CAC scores ≥100 have more than a 4.2‐fold higher risk for a CAD event and 2.8‐fold higher risk for a CVD event compared with those with a CAC score = 0.^16^ A CAC score ≥100 is also associated strongly, and in a graded fashion, with a 10‐year risk of ASCVD (including stroke) above 7.5%.^14^ In the recent 2019 ACC/AHA guidelines on primary prevention of CVD,^40^ CAC score was the only noninvasive test recommended to guide statin prescription in intermediate‐risk patients with a CAC score ≥100.

The role of aspirin in the primary prevention of ASCVD is still controversial.^41^, ^42^ Preliminary findings suggest that CAC score may also help guide decisions for initiating (or deferring) antiplatelet therapy for primary prevention.^26^, ^43^ Recently, the MESA researchers suggested that patients with CAC scores ≥100 may be a good candidates for aspirin therapy for primary prevention,^26^ although the net benefit is expected to be modest. Importantly, in patients with a CAC score of zero, the risk of bleeding appears to be greater than the potential benefit, so aspirin therapy for primary prevention should be avoided. We recently reported that CAC scores are directly related to the risk of a future MI.^44^ The preventive effect of antiplatelet therapy was clearly demonstrated in subjects with CAC scores ≥100, but again, this benefit was partially offset by an increased risk of bleeding.

Even though we found an association between CAC score and the occurrence of future CV events, the role of coronary calcium on the natural history of atherosclerosis remains uncertain. Some researchers suggest that large calcification is a marker of coronary plaque stabilization and thus associated with stable angina (and a reduced risk of acute coronary syndrome events).^45^, ^46^ In our study, we used a calcium score calculated by the Agatston method (which is simple and widely used) during the study period. However, there are novel measures of CAC, such as calcium characteristic, calcium density, and CAC distribution, that may provide improved guidance to management and prevention treatment.^47^, ^48^

Interestingly, the degree of coronary stenosis detected by coronary CT angiography (CCTA) was not predictive of MACEs in our study after adjusting for other covariables. This finding was similar to that of Cho and colleagues who reported that there is no further incremental value gained by the addition of CCTA findings to a model incorporating risk factors and CAC score.^49^ In our population, there were several possible explanations for this counter‐intuitive result. First, the percentage of our subjects with obstructive CAD was only 20.6%. Second, the vast majority of MIs (60%–83%) occur at the site of a nonobstructive plaque.^50^ Thus, CCTA will fail to identify the vast number of asymptomatic patients at risk because an obstructive coronary plaque (>50% stenosis in the artery) is most often not the site of the subsequent cardiovascular event (MI or sudden cardiac death).^51^ Third, there is evidence that CCTA sometimes overestimates CAD severity due to a blooming artifact from coronary calcification.^52^, ^53^ Therefore, the severity of a lesion may have been misclassified. Moreover, our study used 50% of stenosis instead of 70% as the definition of obstructive CAD. This cut‐off might be suboptimal for reflecting the severity of stenosis. The development of functional CCTA, rather than just anatomic imaging, should improve decision‐making regarding revascularization because it assesses flow obstruction and not just anatomy.^54^ Lastly, the concurrent use of antiplatelet or statin therapy might reduce the long‐term occurrence of MACEs.

In summary, the addition of CAC score and CAVI to clinical risk factors should improve the prediction of long‐term occurrence of MACEs and enhance personalized allocation of antiplatelet and statin therapy in primary prevention. The CAC score was a stronger predictor than CAVI and was more reliable than the degree of stenosis as detected by CCTA. CAVI probably is an additional predictor of MACEs, especially in the patients with noncalcified plaques or CAC scores <100. To determine whether they improve the guidance of treatment and prevention of CV events, a future randomized controlled study is needed to evaluate the efficacy of different treatment modalities in each group of patients guided by coronary CT scan findings and/or CAVI.

### 4.1 Limitations of the study

This study had some limitations. First, the analysis did not consider individual patient symptoms at baseline because these data were not completely recorded and unable to adjudicate. Therefore, we could not assess whether predictive values of CAC scores or CAVI on MACEs would be different between asymptomatic and symptom patients. However, most symptoms in our participants (i.e., chest pain on exertion or dyspnea on exertion) were mild. Second, this study did not aim to analyze the effect of concurrent treatments (i.e., antiplatelet, statin, revascularization) on the MACEs. However, to adjust the treatment effect, aspirin and statin therapy were added as covariables and were apparently associated with the higher risk or paradoxical of treatment effects. To clarify this finding, it needs to do propensity score, but this was out of our scope of the study. Other possible explanation of the ineffectiveness of therapy includes discontinuance or no compliance of treatment or including the secondary prevention treatment after CV events occur. Third, follow‐up data were obtained from medical records and government IT data bases, not prospectively planned data collections from patients, and this might have led to some under‐reporting of CV events. Finally, our cohort was not ethically heterogeneous, as all were Asian. Therefore, the discrepancies between studies could be attributed, in part, to ethnic differences.

## 5 CONCLUSION

CAC score and arterial stiffness as reflected by CAVI can enhance risk evaluation beyond that provided by traditional risk factors alone. CAC scores of ≥100 and CAVIs of ≥9.0 predicted the occurrence of long‐term MACEs in both asymptomatic patients with risk factors and symptomatic patients with suspected stable CAD. An advantage of CAVI is that it can be used as a screening tool to predict the CV risk in patients with fibrofatty or noncalcified plaque. However, these two risk predictors must be validated to determine whether they improve the guidance of treatment and prevention of future CV events and prolong survival.

## CONFLICT OF INTEREST

The authors declare no conflict of interest.

## Supporting information

Supplementary information.

## Data Availability

The data support the findings of this study are available from the corresponding author upon reasonable request.
