# Supplementary material for: Predictive values of coronary artery calcium and arterial stiffness for long‐term cardiovascular events in patients with stable coronary artery disease
Source: Clin Cardiol. 2022 Nov 29;46(2):171–83. doi: 10.1002/clc.23955 (PMC9933115; doi:10.1002/clc.23955)
Supplement: Supplementary file 1 — Supplementary information. [file CLC-46-171-s001.docx]

**Supplemental Table 1.** Baseline characteristics of enrolled subjects

| Characteristic | Total |
| --- | --- |
|  | n=8,687 |
| Age (years), mean±SD | 59.0±8.4 |
| Sex (%) |  |
| Male | 36.7 |
| Female | 63.3 |
| BMI (kg/m^2^), mean±SD | 24.9±3.6 |
| Waist circumference (inches), mean (SD) | 34.3±4.2 |
| Smoking status (%) |  |
| Current/Ex-smoker | 13.8 |
| Never smoked | 86.2 |
| Hypertension (%) | 42.3 |
| DM (%) | 13.7 |
| Hypercholesterolemia (%) | 52.5 |
| CKD (eGFR <60) (%) | 7.2 |
| LDL-C (mg/dL), mean±SD | 132.4±37.7 |
| HDL-C (mg/dL), mean±SD | 52.6±13.7 |
| Triglyceride (mg/dL), median (range) | 109 (22, 955) |
| Uric acid (mg/dL), median (range) | 5.2 (1.2, 13.3) |
| CAVI, mean±SD | 8.9±2.2 |
| CAVI >9 (%) | 36.9 |
| CAC score (%) |  |
| ≥ 400 | 6.1 |
| 100- 399 | 12.4 |
| 1- 99 | 31.9 |
| 0 | 49.6 |
| Degree of stenosis (%) |  |
| ≥50% | 20.5 |
| 1% -<50% | 39.3 |
| 0% | 40.2 |
| Number of stenotic vessels (%) |  |
| 3-vessels | 2.8 |
| 2-vessels | 4.8 |
| 1-vessel | 13.7 |
| None | 78.7 |
| LM disease (>50% stenosis) (%) | 1.1 |
| **Concurrent Treatment (%)** |  |
| Antiplatelet | 31.5 |
| Statin | 67.4 |

ABI: ankle-brachial index, ACEI: angiotensin receptor enzyme inhibitor, ARB: angiotensin receptor blocker, BMI: body mass index, CAC: coronary artery calcium, CAD: coronary artery disease, CKD: chronic kidney disease, DM: diabetes mellitus, eGFR: estimated glomerular filtration rate (mL/min/1.73m^2^), HDL-C: high density lipoprotein cholesterol, LDL-C: low density lipoprotein cholesterol, LM: left main.

**Supplemental Table 2.** Long-term clinical outcomes stratified by magnitude of CAC score

| Characteristic | Total | CAC score =0 | CAC score 1-99 | CAC score 100-399 | CAC score >400 | P-value |
| --- | --- | --- | --- | --- | --- | --- |
|  | N=8687 | N=4308 | N=2765 | N=1075 | N=528 |  |
| CV death (%) | 0.7 | 0.4 | 0.5 | 1.3 | 3.0 | <0.001 |
| All causes of death (%) | 4.6 | 2.8 | 4.6 | 9.4 | 10.8 | <0.001 |
| Non-fatal MI (%) | 2.3 | 0.7 | 1.8 | 5.9 | 10.2 | <0.001 |
| Non-fatal stroke (%) | 6.0 | 4.3 | 6.3 | 8.8 | 12.3 | <0.001 |
| MACEs (%) | 8.0 | 5.0 | 7.8 | 13.9 | 20.6 | <0.001 |

CV: cardiovascular, MACEs: major adverse cardiovascular events (CV death, non-fatal MI, or non-fatal stroke)

**Supplemental Table 3.** Long-term clinical outcomes stratified by CAVI

| Characteristic | Total | CAVI <9 | CAVI >9 | P-value |
| --- | --- | --- | --- | --- |
|  | N=8687 | N=5477 | N=3210 |  |
| CV death (%) | 0.7 | 0.3 | 1.5 | <0.001 |
| All causes of death (%) | 5.0 | 3.2 | 8.1 | <0.001 |
| Non-fatal MI (%) | 2.2 | 1.8 | 3.4 | <0.001 |
| Non-fatal stroke (%) | 6.0 | 4.4 | 9.8 | <0.001 |
| MACEs (%) | 7.9 | 5.9 | 12.8 | <0.001 |

CV: cardiovascular, MACEs: major adverse cardiovascular events (CV death, non-fatal MI, or non-fatal stroke)
